# Sclerobanding (Combined Rubber Band Ligation with 3% Polidocanol Foam Sclerotherapy) for the Treatment of Second- and Third-Degree Hemorrhoidal Disease: Feasibility and Short-Term Outcomes

**DOI:** 10.3390/jcm11010218

**Published:** 2021-12-31

**Authors:** Francesco Pata, Luigi Maria Bracchitta, Giancarlo D’Ambrosio, Salvatore Bracchitta

**Affiliations:** 1 Department of Surgery, Nicola Giannettasio Hospital, 87064 Corigliano-Rossano, Italy; 2 La Sapienza University, 00185 Rome, Italy; 3 Primary Care Department, ATS Città Metropolitana, 20100 Milan, Italy; luigimaria.bracchitta@gmail.com; 4 Department of General Surgery, Surgical Specialties and Organ Transplantation, La Sapienza University, 00161 Rome, Italy; giancarlo.dambrosio@uniroma1.it; 5 Coloproctology Centre, Clinica del Mediterraneo, 97100 Ragusa, Italy; dott.salvatorebracchitta@gmail.com

**Keywords:** hemorrhoids, sclerobanding, rubber band ligation, sclerotherapy, hemorrhoidal disease, HD, polidocanol, haemorrhoidal disease, hemorrhoids, proctology, foam

## Abstract

Background: Sclerobanding is a novel technique combining rubber band ligation with 3% polidocanol foam sclerotherapy for the treatment of hemorrhoidal disease (HD). The aim of this study is to evaluate the feasibility, safety and short-term outcomes of sclerobanding in the treatment of second- and third-degree HD. Methods: A retrospective analysis of second- and third-degree HD cases from November 2017 to August 2021 was performed. Patients on anticoagulants or with other HD degrees were excluded. Follow-up was conducted at 1 month, 3 months, 6 months, 1 year and then every 12 months. Results: 97 patients with second- (20 pts; 20.6%) and third-degree (77 pts; 79.4%) HD with a mean age of 52 years (20–84; SD ± 15.5) were included. Fifty-six patients were men (57.7%) and forty-one women (42.3%). Median follow-up was 13 months (1–26 months). No intraoperative adverse events or drug-related side effects occurred. Minor complications occurred in four patients (4.1%) in the first 30 postoperative days and all resolved after conservative treatment at the 3-month follow-up visit. No mortality or readmissions were observed. Conclusions: Sclerobanding is a safe technique with a low rate of minor postoperative complications. Further studies on larger samples are necessary to establish the effectiveness and long-term outcomes of the technique.

## 1. Introduction

Despite the availability of several procedures, the current management of hemorrhoidal disease (HD) is still controversial, especially in the middle degree of HD not responsive to conservative treatment. Stapled hemorrhoidopexy (SH) is declining due to higher recurrence rate and the risk of rare but severe complications [1,2]. Distal Doppler-guided transanal hemorrhoidal dearterialization with mucopexy (THD) seems associated with decreased postoperative pain and fast recovery in comparison with SH and hemorrhoidectomy, but shows higher pain and higher rate of bleeding requiring transfusion in comparison with rubber band ligation (RBL) [3,4]. Furthermore, these techniques require dedicated devices, are performed under general or spinal anesthesia with increased costs and need admission to the hospital.

The COVID-19 pandemic has added further issues, due to the cancellation of elective lists and reduction of outpatient clinics [5,6,7], with coloproctological cases often neglected or treated in advanced stages [8,9].

RBL and injection sclerotherapy (SCT) are office-based effective procedures in the treatment of second- and third-degree HD with reduced costs and a low complication rate [3,10,11,12,13]. However, both present some complications, such as late bleeding in RBL or the risk of mucosal ulceration, prostatic abscess or acute prostatitis in SCT.

Sclerobanding is a novel technique, recently described in the literature [14], aiming to merge the advantages of sclerotherapy with 3% polidocanol foam with rubber band ligation to treat second- and third-degree HD. The procedure can be performed on an outpatient basis without local anesthesia, reduced costs and no hospitalization or anesthetic support required.

The aim of this study is to establish the feasibility, safety and early outcomes of sclerobanding in a cohort of patients with symptomatic second- and third-degree HD, unresponsive to conservative treatment.

## 2. Materials and Methods

This was a retrospective study and was conducted according with the STROBE (Strengthening the Reporting of Observational Studies in Epidemiology (STROBE) statement for cohort studies) guidelines [15].

Between November 2017 and August 2021, 125 patients underwent sclerobanding at the coloproctology center “Clinica del Mediterraneo” in Ragusa, Italy. Demographic, perioperative data and postoperative complications (after 30 postoperative days), were recorded in a local database. Each patient consented to the procedure and signed a written consent form.

Goligher classification was used to stage the disease. Inclusion criteria were any patient aged ≥ 18 years with second- and third-degree HD unresponsive to conservative treatment. Patients under anticoagulant therapy or patients not presenting at follow-up appointments were excluded.

All procedures were performed by the same colorectal surgeon. Patients were followed up with in clinic after 1 week, 4 weeks, 3 months, 6 months and 12 months. Evaluation consisted of an interview to detect symptoms suggesting recurrence (bleeding, prolapse, discomfort/anal burning), inspection of the anorectal region in Sims position and digital rectal examination. An anoscopy was performed starting at the 3-month visit.

The primary aim of the study was to evaluate the feasibility and safety of sclerobanding expressed as a percentage of intraoperative and postoperative adverse events occurring in the first 30 days.

The secondary aim was to assess if any difference in terms of intraoperative adverse events and complication rates occurred in the two groups (second-degree vs third-degree) identified by the Goligher classification.

### 2.1. Surgical Technique

We have described sclerobanding in a recent publication [14]. No antibiotic is administered before the procedure. No anesthesia is necessary, but in some cases local or local-regional anesthesia may be required for anxious patients. The patient is placed in the lithotomy position to gain the best view of the anorectal region and to confirm the preoperative staging. Each hemorrhoidal nodule is ligated at the basis above the dentate line by a rubber band. Any suspicious area can be biopsied and sent for histology. Subsequently, 2 or 3 mL of 3% polidocanol foam, obtained as described by Moser [16], is injected into the ligated nodule. After the procedure, the patient is monitored for 1 h (or 1 h after the return of motor function in case of spinal anesthesia) and then discharged with a dedicated phone number in case of urgent needs. Analgesics and stool softeners are prescribed in case of pain or in patients with chronic constipation.

**Figure 1:**
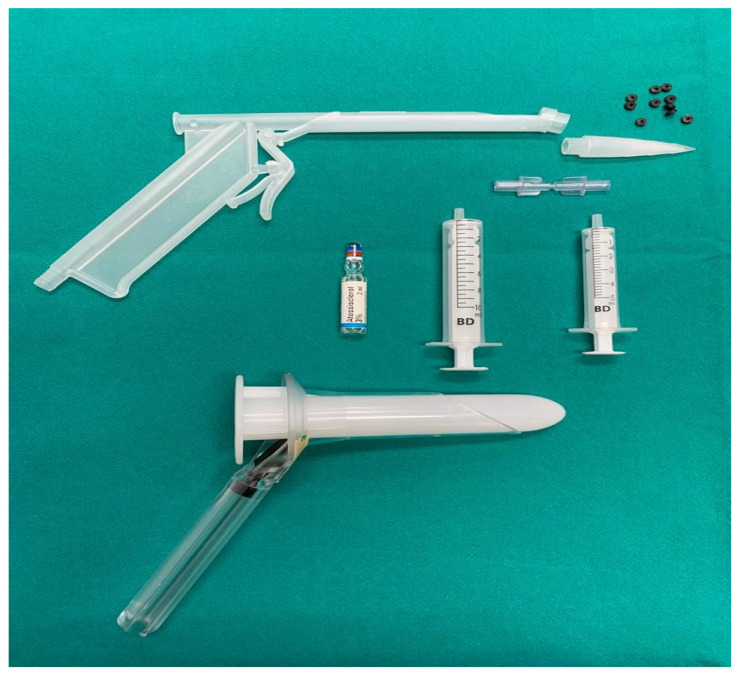
Sclerobanding armamentarium for a single procedure.

Figure 1 shows the armamentarium needed to perform the procedure.

### 2.2. Statistical Analysis

Data are presented as means ± standard deviation, range or percentage. The Fisher Exact Test was used to compare the complication rate between second- and third-degree HD groups. A *p*-value < 0.05 was considered statistically significant.

Statistical analysis was performed using Microsoft**^®^** Excel**^®^** 2016 (Microsoft Corporation, Redmon, WA, USA).

## 3. Results

A total of 97 patients with second- (20 pts; 20.6%) and third-degree (77 pts; 79.4%) HD with a mean age of 52 (20–84; SD ± 15.5) years were included. Fifty-six patients were male (57.7%) and forty-one patients were women (42.3%). No intraoperative adverse events or drug-related side effects occurred. All patients were discharged 1 h after the procedure (1 h after the return of motor function in case of spinal anesthesia). The procedural results are detailed in Table 1.

At the 1-month follow-up, minor complications occurred in four patients (4.1%): three cases of thrombosis of a minor hemorrhoidal nodule, treated by lifestyle modifications and oral flavonoids, and one case of defecation urgency, treated with a suggestion of methylcellulose by mouth for two weeks. All complications were detected in the third-degree group and were resolved at the 3-month follow-up visit. No readmissions or major complications were registered. The 30-day complication rate was 5.2% in the third-degree group versus zero in the second-degree group, but the difference was not statistically significant (Fisher exact test statistic value 0.5578 *p* > 0.05).

Ten patients (10.3%) underwent spinal anesthesia, forty-six (47.4%) required local anesthesia and forty-one did not require any anesthesia (Table 2).

## 4. Discussion

Our study showed that sclerobanding is a safe technique with a low complication rate and satisfying short-term outcomes.

Although newest techniques are currently available in clinical practice, SCT and RBL remain the most solid options in the treatment of second- and third-degree HD, with a high grade of evidence [3,17,18,19]. Both techniques can be performed in an outpatient clinic without anesthesia, are repeatable and sphincter-saving, thus not associated with chronic sequalae such as urgency, tenesmus and fecal incontinence.

RBL was first described by Baron in 1963 [20].The fundamental steps of the procedure have not changed, although disposable devices, ligators with a suction channel (to avoid the need of an assistant) and the endoscopic approach have been introduced to improve efficacy [21]. A silicone band is applied at the base of the hemorrhoids above the dentate line [22] to avoid pain and drops out after 7–10 days.

RBL is effective in first- to third-degree HD with an improvement of symptoms in 78–100% of cases in different studies [3,17]. Complications are usually mild and transient although case of pelvic abscess and sepsis with death have been anecdotally reported [10,23]. Significative bleeding, occasionally requiring transfusion and admission in the hospital, is the most feared complication [10]. It usually occurs between the 10th and the 14 postoperative day with an incidence of 1.7–2.5%, most frequently in patients on anticoagulants [23,24].

Sclerotherapy, with the introduction of polidocanol foam, is now emerging as a safe and effective technique with an overall success rate of 78%, increasing to 86% after a second session [25]. However, RCT and long-term follow-up are lacking.

Sclerobanding merge both techniques with the aim of increasing effectiveness and reducing some typical complications of both procedures. Sclerotherapy of the ligated nodule aims to increase the inflammatory reaction and the “lifting effect” on the mucosal prolapse and avoid the delayed bleeding, sometime severe, reported in literature [26]. On the other hand, the spreading of the 3% polidocanol after the ligation of the nodule by the rubber band represents a barrier to the diffusion of the foam in the submucosa, a potential cause of severe pain, mucosal ulceration, abscess and acute prostatitis reported in some series [16,27,28]. In our study no case of intraoperative or postoperative bleeding or septic complications were detected in line with the rationale of the technique.

The COVID-19 pandemic represents an additional challenge [29,30]. The extended wait times for elective surgeries and the backlog caused by the repeated lockdowns during pandemic waves is an unwanted heritage of the SARS-CoV-2 outbreak [31,32]. It has been estimated that any further week of service disruption will result in an additional burden of 2,367,050 operations [6].

Sclerobanding may present promising features in post-pandemic surgical recovery plans: it can be performed in-clinic; as an office procedure, it does not require anesthesia or a formal operating room and presents a low risk of readmission and complications. In the present study, ten patients underwent spinal anesthesia, but this was mainly related to a specific patient request rather than being for clinical reasons. A significant number of HD patients may be managed by this technique in an outpatient setting, reducing the backlog due to proctological diseases and the associated healthcare costs.

The total cost of a sclerobanding armamentarium for a single procedure is around EUR 30 with a more significant impact due to the absence of hospitalization, healthcare resource consumption in the operating theater and rapid return to work and daily activities.

Five studies [33,34,35,36,37], mainly retrospective (Table 3), have been published in the literature about the concomitant use of SCT with RBL in the treatment of HD, but with different technical details and omitting important information for a fair comparison. All authors used oil-based sclerotizing agents, often injected into the submucosal plane. Chew et al. reported a reverse approach, with SCT firstly applied in the hemorrhoidal nodule to facilitate the banding [33]. Kanellos et al. both in their observational study [34] and in their prospective randomized trial [35] ligated larger hemorrhoidal nodules with a rubber band, injecting the sclerosant agent into the minor nodules.

In our study, the complication rate was low (4.1%) and represented by only minor self-limiting complications (three cases of a thrombosed hemorrhoidal nodule and one case of urgency). All complications disappeared at the 3-month follow-up visit and were registered in the third-degree HD group, but, when compared with the second-degree HD group, this difference was not statistically significant (*p* > 0.05). No major complications, readmission or mortality occurred in both groups.

This study presents some limitations. It is a single center study with all procedures performed by the same colorectal surgeon with all potential biases inherent to this study design. No comparison with other treatments has been performed and long-term results are lacking. However, both techniques on which sclerobanding is based have been deeply analyzed in other studies. The absence in the present study of typical, albeit rare, complications related to both techniques (such as tardive bleeding or infection) suggests that sclerobanding may present further advantages in comparison with both techniques performed separately.

## 5. Conclusions

Sclerobanding is a safe and low-cost technique for the treatment of second- and third-degree hemorrhoidal disease. The procedure can be performed in an outpatient clinic and is repeatable. However, due to the inherent limitations of the study design, multicentric and comparative studies and a longer follow-up are necessary to demonstrate its superiority in comparison with other techniques.

## Figures and Tables

**Table 1:** Demographic and postoperative outcomes of the patients included in the study. Age and ligations per procedure are expressed as a mean with therange between parentheses. Median is expressed as a median.

VARIABLE	N (%)
**Patients**	97
Man	56 (57.1%)
Women	42 (42.9%)
**Age**	52 years (20–84)
**Goligher classification**	
2nd degree	20 (20.4%)
3rd degree	78 (79.6%)
**Ligations per procedure**	2.7 (1–3)
**Follow-up (median)**	13 months (1–26)
**Intraoperative complications**	0 (0%)
**30-day complications**	4 (4.1%)
2nd degree	0 (0%)
3rd degree	4 (5.2%)
**Readmission**	0%
**Mortality**	0%

**Table 2:** Anesthesia techniques performed in the cohort of the 97 patients included in the study.

Anesthesia Techniques	No. of Patients (%)
No anesthesia	41 (42.3%)
Local anesthesia	46 (47.4%)
Spinal anesthesia	10 (10.3%)

**Table 3:** Published studies reporting concomitant use of sclerotherapy and rubber band ligation to treat hemorrhoidal disease (HD).

Authors	Year	Design	No. of Patients	Technique	Degrees of HD	Follow-Up	Overall Complications	Recurrence
Rabau et al. [36]	1985	Retrospective	178	RBL (first) then SCT of the same nodule	I to III	1 year	5.6%	10–15%
Choi et al. [37]	1985	Retrospective	111	RBL (first) then SCT of the same nodule	I to III	18 months (range 2–60)	1.8% (8.1% considering postoperative pain in the first 72 h)	15%
Kanellos et al. [34]	1999	Prospective	83	RBL for larger and SCT for minor nodules	II	2 years	9.2%	NR
Chew et al. [33]	2003	Retrospective	1102	Sclerotherapy (first) then RBL of the same nodule	I to II	1–11 years (mail/phone interview)	3.1%	16%
Kanellos et al. [35]	2003	Randomized control trial	255 (85 in RBL + SCT group)	RBL for larger and SCT for minor nodules	II	4 years	10.8%	10%

## Data Availability

The data presented in this study are available on request from the corresponding author. The data are not publicly available due to privacy rules and the need of a new approval process for sharing them with third parties.
