# Correction: Pata et al. Sclerobanding (Combined Rubber Band Ligation with 3% Polidocanol Foam Sclerotherapy) for the Treatment of Second- and Third-Degree Hemorrhoidal Disease: Feasibility and Short-Term Outcomes. *J. Clin. Med.* 2022, *11*, 218

**DOI:** 10.3390/jcm12155159

**Published:** 2023-08-07

**Authors:** Francesco Pata, Luigi Maria Bracchitta, Giancarlo D’Ambrosio, Salvatore Bracchitta

**Affiliations:** 1 Department of Surgery, Nicola Giannettasio Hospital, 87064 Corigliano-Rossano, Italy; 2 La Sapienza University, 00185 Rome, Italy; 3 Primary Care Department, ATS Città Metropolitana, 20100 Milan, Italy; luigimaria.bracchitta@gmail.com; 4 Department of General Surgery, Surgical Specialties and Organ Transplantation, La Sapienza University, 00161 Rome, Italy; giancarlo.dambrosio@uniroma1.it; 5 Coloproctology Centre, Clinica del Mediterraneo, 97100 Ragusa, Italy; dott.salvatorebracchitta@gmail.com

## 1. Table Legend

In the original publication [1], there was a mistake in the legend for Table 1. “therange” should be changed to “the range”, and the sentence “Median is expressed as a median” should be changed to “Follow-up is expressed as a median”. The correct legend appears below.

**Table 1.** Demographic and postoperative outcomes of the patients included in the study. Age and ligations per procedure are expressed as a mean with the range between parentheses. Follow-up is expressed as a median.

## 2. Error in Table

In the original publication [1], there was a mistake in Table 1 as published. “Man” should be changed to “Men”, “56 (57.1%)” should be changed to “56 (57.7%)”, 42 (42.9%) should be changed to “41 (42.3 %)”, “20 (20.4%)” should be changed to “20 (20.6%)”, and “78 (79.6%)” should be changed to “77 (79.4%)”. The corrected Table 1 appears below.

The authors state that the scientific conclusions are unaffected. This correction was approved by the Academic Editor. The original publication has also been updated.

## Figures and Tables

**Table 1:** Demographic and postoperative outcomes of the patients included in the study. Age and ligations per procedure are expressed as a mean with the range between parentheses. Follow-up is expressed as a median.

VARIABLE	N (%)
**Patients**	97
Men	56 (57.7%)
Women	41 (42.3%)
**Age**	52 years (20–84)
**Goligher classification**	
2nd degree	20 (20.6%)
3rd degree	77 (79.4%)
**Ligations per procedure**	2.7 (1–3)
**Follow-up (median)**	13 months (1–26)
**Intraoperative complications**	0 (0%)
**30-day complications**	4 (4.1%)
2nd degree	0 (0%)
3rd degree	4 (5.2%)
**Readmission**	0%
**Mortality**	0%
